# A Dozen Years of Baleen Hormones: Validations, Discoveries, Surprises, and Next Steps

**DOI:** 10.1093/icb/icag063

**Published:** 2026-06-08

**Authors:** Kathleen E Hunt, Jennifer Jelincic, Nadine Lysiak, Raphaela Stimmelmayr, Allison Case, Rebecca Evey, Alejandro Fernández Ajó, Danielle Dillon, Joshua Reed, Leslie New, C Loren Buck

**Affiliations:** Marine Mammal Institute, Oregon State University, Newport, OR 97365, USA; Department of Biology, George Mason University, Fairfax, VA 22030, USA; Marine Mammal Institute, Oregon State University, Newport, OR 97365, USA; Anderson Cabot Center for Ocean Life, New England Aquarium, Boston, MA 02110, USA; North Slope Borough Department of Wildlife Management, Utqiagvik,AK 99723, USA; Department of Biology, George Mason University, Fairfax, VA 22030, USA; Department of Biology, George Mason University, Fairfax, VA 22030, USA; Marine Mammal Institute, Oregon State University, Newport, OR 97365, USA; Anderson Cabot Center for Ocean Life, New England Aquarium, Boston, MA 02110, USA; Department of Mathematics, Computer Science and Statistics, Ursinus College, Collegeville, PA 19426, USA; Department of Mathematics, Computer Science and Statistics, Ursinus College, Collegeville, PA 19426, USA; Department of Community, Environment, and Policy, Mel and Enid Zuckerman College of Public Health, University of Arizona, Tucson, AZ85724, USA

## Abstract

Since the first publication in 2014 reporting that progesterone is detectable in whale baleen, numerous studies have confirmed that patterns of hormones in baleen can provide a multiyear time series of continuous endocrine information from individual whales. The field is poised to expand substantially in the near future, and thus it is an opportune time to review findings and identify current knowledge gaps and pathways for future research. A search of baleen-hormone literature reveals 30 publications that, in total, investigate eight steroid hormones and two thyroid hormones in baleen of 10 species of mysticete whale, with the pygmy right whale representing the only mysticete family not yet studied. An early phase of methodological validations optimized the technique, including reduction of sample mass requirement and improvements in hormone yield. Steroid hormones have consistently passed technical and physiological validations; thyroid hormones, however, are still in need of physiological validations. Later literature has entailed a series of descriptive studies, which typically combine endocrine and isotope analyses to elucidate typical hormone ranges and patterns across years in relation to species, sex, age class, and reproductive state. Most descriptive studies have been limited to a small *n* of individuals (a consequence of the high *n* of subsamples per whale), yet have been highly informative nonetheless, revealing many unexpected findings (e.g., evidence suggestive of extended gestation, timing and location of breeding, reproductive senescence, early sexual maturity, pregnancy loss, and effects of stressors). Such case-study reports remain of considerable value, but the field is increasingly expanding to include hypothesis-driven research on ecological questions of broad significance, such as influences of oceanographic factors and anthropogenic stressors, and the physiological and behavioral plasticity of individual responses to such environmental drivers. Addressing such questions will require robust statistical frameworks and larger sample sizes of individual whales, a daunting task given that a single baleen specimen can generate > 150 samples requiring months of labor and associated costs. Thus, increased collaborations could be both fruitful and necessary (e.g., a baleen-hormone research consortium wherein datasets can be pooled across research teams). In sum, baleen hormone research has provided unique and invaluable insights into patterns of physiology across time in the great whales, and has great promise to continue advancing understanding of the biology of these vulnerable, long-lived, enigmatic species.

The mysticete (baleen-bearing) whales have long been one of the most poorly understood groups of mammals. Many biological traits of mysticetes remain unknown or only roughly estimated, despite their ecological importance as keystone species and predators, their unique biological traits, their continued need for effective conservation and management, and high public interest. Data gaps often include, for example, fundamental life history traits such as gestation length, intercalving interval, reproductive seasonality, age of sexual maturity, occurrence of senescence, and location of breeding grounds (Hunt et al. 2013; Eichenberger et al. 2023). These gaps in knowledge are due to the logistical challenges of studying live whales at sea, such as vast home ranges, lengthy annual migrations, remote offshore habitat, difficulty in locating and observing individuals, and lack of practical methods for live-capture and sampling. Creative applications of novel tagging technologies, biopsies, acoustics, drone-based imaging, and shipboard photography have partly filled these data gaps (e.g., Pettis et al. 2004; Kellar 2008; Hunt et al. 2013; Stafford 2022; Bierlich et al. 2024), but despite such advances, key information on reproductive, stress, and metabolic physiology remains limited. Specifically, it has become clear that new methods of assessing hormonal patterns as a means to assess physiology and infer behavior would be especially useful, particularly methods that allow for within-individual longitudinal assessment of hormones (i.e., repeated measures).

Several decades of terrestrial research have revealed that the vertebrate steroid and thyroid hormones are detectable in a wide variety of non-plasma sample types, which in whales can include fecal samples, blubber and skin biopsies, respiratory vapor, and epidermal scrapings (Mello et al. 2016; Burgess and Lanyon 2026). These sample types have proven informative, yet it remains difficult to collect enough samples over time (e.g., within-individual repeated measures) to investigate questions regarding reproductive cycles and important life-history transitions. Two mysticete sample types, however, contain a “time series” of successive layers deposited over time: earwax plugs (cerumen), and baleen. Both of these sample types can be collected only once a whale has died (e.g., from necropsies), but provide retrospective data on the whale’s physiological status during life. Earwax plugs have the advantage of capturing the entire lifetime of the animal (e.g, Trumble et al. 2013 ), but have proven to have relatively poor temporal resolution (∼6–12 months represented per sampling point), which obscures important detail on timing of physiological events. Further, earplugs are rarely collected, as collection requires anatomical dissection of the external auditory meatus; additionally, given their wax composition they are very fragile and do not form well in all mysticete species. Hence, research on this sample type has remained limited. In contrast, baleen, which consists of long strips of keratinous tissue in the mouths of mysticete whales, is readily collectable from deceased whales. Ample baleen sample archives exist from both the commercial whaling era and from modern times. Today, baleen is routinely collected during necropsies of stranded whales (e.g., Lysiak et al. 2018) as well as from whales harvested in indigenous subsistence hunts (Carroll 1976; Higdon 2010). Thus, considerable archives of baleen exist for study, providing a potential solution to the long-standing problem of studying hormones across time in the mysticete whales, both within and across individuals.

Baleen is a keratin tissue that grows from the epidermis of the upper palate of mysticete whales, forming their filter-feeding apparatus (Werth and Potvin 2024; Fig. 1). Classed as a cornified keratinized epidermal tissue (Bragulla and Homberger 2009), baleen consists of hundreds of long vertical pieces (“plates”) that hang in parallel much like curtains on the right and left sides of the mouth. Each baleen plate is an elongated, flat structure that narrows to a pointed tip at the distal, inferior, end, containing numerous closely packed parallel strands of alpha-keratin tubules (aka “horn tubules”; likely mammalian hair) encased in a firm, flexible, matrix of keratin and calcium salts that is reminiscent of fingernail or hoof (Werth 2018; Fig. 1). Like keratin tissues of many other mammals, baleen plates grow slowly and continuously from a “base” region embedded in well-vascularized soft tissue (known as the *Zwischensubstanz*; Fudge et al. 2009; Werth 2018) of the upper palate, accumulating various hormones and other biomarkers from circulation as it grows, and simultaneously wearing away at the distal tip. The progressive erosion of the tip of the plate means that for adults, baleen does not capture the full life of the whale, but rather just the most recent years of life. This timespan may be anywhere from the last ∼1.5 years of life in those species with short baleen (e.g., gray whale, *Eschrichtius robustus*; Fernández Ajó et al. 2024) to the last ∼20 years of life in species with longest baleen (bowhead whale*, Balaena mysticetus*; Hunt et al. 2022; Jelincic et al. 2026a), depending on species-specific baleen length, baleen growth rate (BGR), and age. However, in young individuals (calves and juveniles) in which the distal tip of the plate has not yet begun to erode, a baleen plate may represent the entire life of the animal. Additionally, baleen has good temporal resolution that enables separation of seasons within a year, and hence can support examination of annual cycles and seasonal phenomena (e.g., Hunt et al. 2022), as well as relative timing of stressors and reproductive events.

**Fig. 1:**
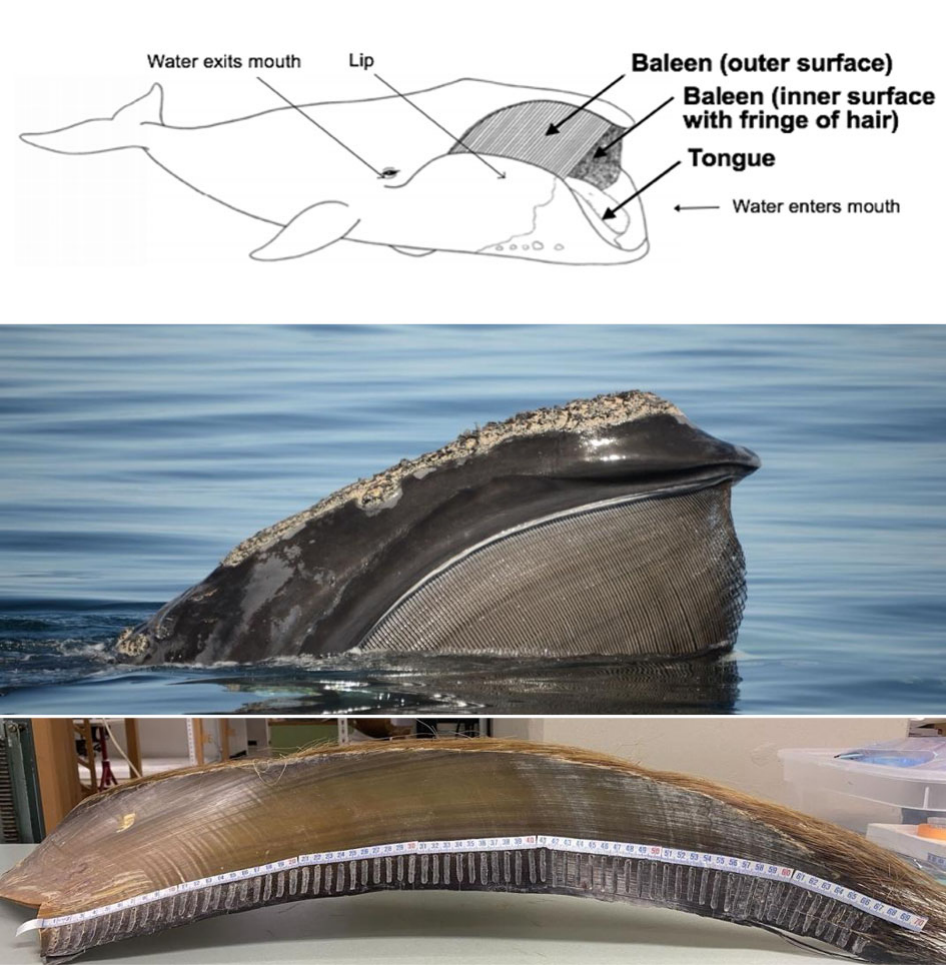
Whale baleen. Top, illustration of a whale showing position of baleen in the mouth. Middle, rostrum of a skim-feeding North Atlantic right whale, *Eubalaena glacialis*; parallel lines are edges of adjacent baleen plates. Bottom, A single baleen plate of a fin whale, *Balaenoptera physalus*, showing measuring tape (affixed with 0 cm = proximal-most point of base of plate, on left) and typical sampling grooves drilled every 1 cm with a hand-held rotary grinder; note hair-like fringe on buccal edge (at top of photo). Top panel modified from Werth (2013) and used with permission; center panel: E Burgess/New England Aquarium (2011), NMFS permit #14233, used with permission; bottom panel: K Hunt (2022) .

Baleen contains not only hormones but also other biomarkers of interest, including stable isotopes derived from the whale’s food sources (Schell et al. 1989; Best and Schell 1996). When examined across the length of a baleen plate, ratios of stable isotopes (^15^N/^14^N and ^13^C/^12^C) often present annual cycles representing successive years (i.e., migration cycles), thus providing the timeline via which hormone data can be interpreted (e.g., Lysiak et al. 2018, Lowe et al. 2022; example in Fig. 2). The importance of these isotope-derived timelines for endocrine studies cannot be overstated; isotope data can reveal key temporal information such as BGR, number of years of baleen growth represented by a given baleen specimen, identification of regions of baleen grown in specific seasons (e.g., summer vs. winter), as well as estimation of important temporal aspects of reproduction (e.g., duration of gestation, intercalving interval, annual testosterone cycles, age of weaning, age of sexual maturity) and stress physiology (e.g., acute vs. chronic causes of death, duration of entanglement events). Thus, almost all endocrine studies of baleen either include isotope analyses for all specimens, or rely heavily on previously published isotope data. As an additional benefit, other aspects of isotope data also provide information relevant for foraging ecology, species distribution and migration (location of foraging, prey selection, trophic level, etc.), with compound-specific analyses revealing even more detail. In addition to isotopes, numerous other analytes are present in baleen as well. For example, baleen has been shown to accumulate mercury (Lowe et al. 2022b; Hobson et al. 2004) and other toxicants (e.g., per- and polyfluoroalkyl substances, PFAS; Savoca et al. 2024) that can deleteriously impact whale health, suggesting potential to explore toxicant burden in relation to physiological parameters such as reproduction.

**Fig. 2:**
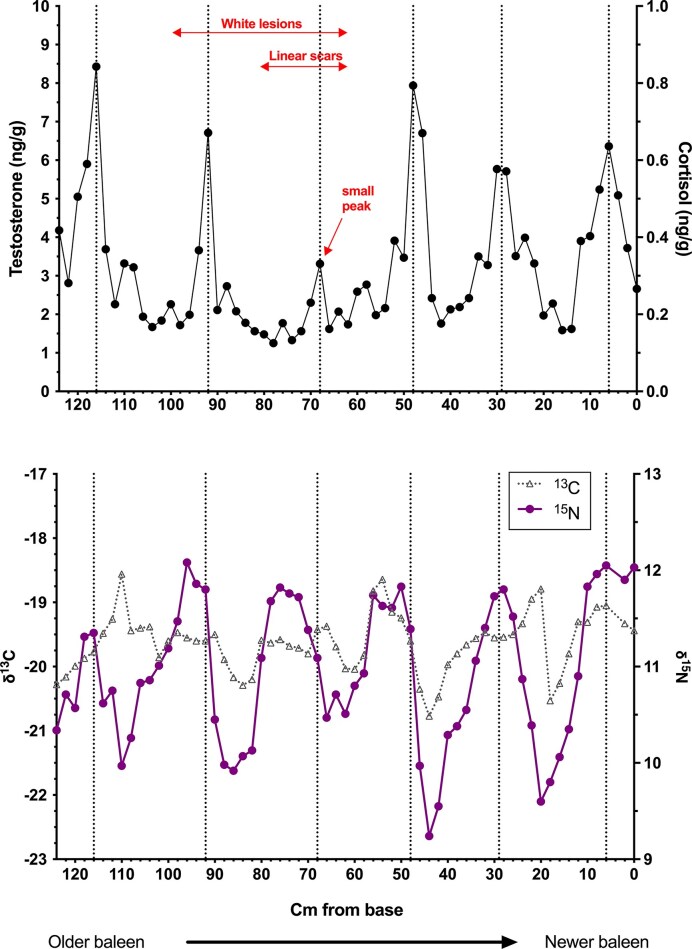
Example of the time series nature of baleen data, evident from hormone data (testosterone, top) and stable isotope data (bulk carbon and nitrogen, bottom) from the same baleen plate from a male North Atlantic right whale. Note regularity and matching periodicity of testosterone cycles and stable isotope cycles, indicating that this baleen specimen contains a time series of six successive years. Arrows at top indicate known physiological stressors, co-occurring with reduced testosterone. From Hunt et al. (2018), used with permission.

Since 1989, whale baleen has largely been studied in the context of addressing questions of foraging ecology using the aforementioned stable isotope analyses (e.g., Schell et al. 1989; Best and Schell 1996; Matthews and Ferguson 2015). However, in the early 2000s, endogenous hormones were reported to be detectable in vertebrate keratin tissues, first in mammalian hair (Davenport et al. 2006) and secondly bird feathers (Bortolotti et al. 2008), with concentrations that represent the animal’s endocrine state at the time when the hair or feather was growing. These discoveries spurred investigation into endocrine analysis of other mammalian keratinized tissues such as spines, vibrissae, horns, nails, claws, and hoof (e.g., Warnock et al. 2010; Peric et al. 2013; Gundlach et al. 2014), as these tissues are related to mammalian hair. In 2014, progesterone was detected in bowhead whale baleen, with patterns suggestive of past pregnancies (Hunt et al. 2014), suggesting that baleen could provide a much-needed method for studying patterns of hormones across time in individual large whales. Numerous additional studies have since been published (see below), and the field appears poised for expansion in the near future. Here, we review the existing literature concerning patterns of hormones in whale baleen, summarizing validation results, unexpected findings, and potential next steps.

## Survey of baleen hormone literature

To locate relevant literature, we performed a search (Google Scholar and PubMed, March 1, 2026, utilizing keywords “baleen” and [“hormone*” or “endocrin*”]), followed by secondary searches of all citations of, and by, the results of the first search. This literature search revealed 30 publications concerning baleen hormones (Table 1). Publication rate has varied from one to four papers per year, increasing to at least eight in the current year, and the authors are aware of over a dozen manuscripts in preparation.

**Table 1:** Summary of peer-reviewed literature on patterns of hormones in whale baleen.

Year	First author	Species^a^	*n* (whales)	Hormones^b^	Primary questions
2014	Hunt	Bowhead	16	F, P4	Pregnancy status (assessed at base of plate only)
2015	Hirt	Bowhead	15	P4	Pregnancy, age effects
2016	Hunt	NARW	2	P4	Pregnancy, intercalving interval, entanglement stress
2017a	Hunt	Bowhead, NARW, blue, fin, humpback, sei, gray, minke	8 (1 per species)	Ald, B, F, E2, P4, T, T3, T4	Assay validations (parallelism, accuracy)
2017b	Hunt	NARW	2	B, F, P4	Stress in relation to pregnancy and inferred entanglement
2018	Hunt	NARW, blue, bowhead	3 (1 per species)	T	Male T cycles, seasonal breeding
2018	Fernández Ajó	SRW, NARW	5 (four SRW, 1 NARW)	B, F	Acute vs. chronic cause of death, chronic stress during unusual mortality event, wounding stress
2018	Lysiak	NARW	1	B, F, E2, P4, T3, T4	Fatal entanglement, pregnancy
2019	Rolland	Bowhead	1	B, F	Chronic stress (entanglement)
2020	Fernández Ajó	SRW	36	B, F, T3	Chronic stressors and relation to wounding stress
2021	Dillon	Bowhead, blue, SRW	3 (1 per species)	B	Methods optimization (keratinase)
2021	Fernández Ajó	SRW	1	P4	Methods optimization (sample mass, solvent:sample ratio)
2021	Gabriele	Humpback	1	B, F, T	Chronic stress of disease, moribund pattern
2021a	Lowe	Humpback	4	P4	Pregnancy
2021b	Lowe	Humpback	4	B, F	Entanglement stress, nutritional stress
2022	Hunt	Bowhead	9	T	Male T cycles, sexual maturity, senescence/age effects
2022a	Lowe	Humpback	2	B, F, E2, T	Reproduction-related stress, age effects, T in females, ovulation, parturition
2023	Lysiak	Bowhead	10	B, E2, P4	Pregnancy, sexual maturity, gestation length, calving interval
2024	Fernández Ajó	Gray	5	F, T3	Baleen growth rate, cortisol, chronic stress during unusual mortality event
2025	Hudson	Bowhead	8	B, T, T3	Patterns in T3 in relation to stress
2025	Shuttleworth	SRW	4	E1, B, F, P4, T	Pregnancy, gestation length, calving interval, parturition
2026	Brown	Humpback	11	B, F	Entanglement stress, injury stress, hormone correlations
2026	Evey	Rice’s	7	B, F, P4, T	Pregnancy, reproductive seasonality, acute vs. chronic stress, patterns in calves
2026a	Jelincic	Bowhead	4	B, DHEA, F, P4, T, T3	Pregnancy, T cycles, environmental change, hormone correlations
2026b	Jelincic	Bowhead	4	B, DHEA, F, P4, T, T3	Male T cycles, seasonal breeding, environmental change
2026c	Jelincic	Bowhead	6	B, DHEA, F, P4, T, T3	Pregnancy, calf loss, calving interval, environmental change
2026	Matthews	Bowhead	19	B, T3	Nutrition-related stress, climate change, environmental effects
2026	de Mello	Sei	11	B, F, P4	Pregnancy, calving interval, reproduction-related stress, gestation length
2026	New	Bowhead	N/A	N/A	Statistical techniques
2026	Reed	Bowhead	N//A	N/A	Statistical techniques

a Bowhead whale, *Balaena mysticetus;* NARW, North Atlantic right whale, *Eubalaena glacialis;* SRW, southern right whale, *Eubalaena australis*; blue whale, *Balaenoptera musculus;* fin (finback) whale, *Balaenoptera physalus;* humpback whale, *Megaptera novaeangliae*; gray whale, *Eschrichtius robustus*; sei whale, *Balaenoptera borealis;* Rice’s whale, *Balaenoptera ricei*; minke whale, *Balaenoptera acutorostrata*.

b Ald = aldosterone, B = corticosterone, DHEA = dehydroepiandrosterone, E1 = estrone, E2 = estradiol, F = cortisol, P4 = progesterone, T = testosterone, T3 = tri-iodothyronine, T4 = thyroxine.

### Species

Hormones are detectable in baleen of at least 10 species, including 3 balaenids (bowhead; North Atlantic right, *Eubalaena glacialis;* southern right, *E. australis*), and 7 rorquals (gray; humpback, *Megaptera novaeangliae*; blue, *Balaenoptera musculus*; fin, *B. physalus*; sei, *B. borealis;* Rice’s, *B. ricei*; and minke, *B. acutorostrata*; Table 1). Research has not been evenly distributed across species; rather, baleen-hormone research began with, and still focuses on, the balaenids (which have longest baleen, and also some of the most comprehensive sightings records and necropsy data, including gonadal histology and age estimation methods; e.g., George and Thewissen 2021), specifically bowhead whales (15 papers), North Atlantic right whales (6 papers), and southern right whales (5 papers) (Table 1). Rorquals, however, are now increasingly being studied, with results presented in six publications on humpbacks, three involving blue whales, two publications each for gray and sei whales, and single publications on minke and Rice’s whales (Table 1). While most research has focused on species with very long (*Balaenidae*) or moderate length (most rorquals) baleen, publications on gray and Rice’s whales (∼1.5 y timeline), as well as southern right whale calves (<6 month timeline) demonstrate that even short baleen can provide useful information (Fernández Ajó et al. 2018, 2020, 2024; Evey et al. 2026). Remaining untested species largely consist of close relatives of whale species already studied (e.g., North Pacific right, *E. japonica*, close relative of the North Atlantic right), the minke whale (for which assay validations have been performed but no detailed case studies have yet been published), and the pygmy right whale (*Caperea marginata*), the latter species representing the only mysticete family still unstudied (variously *Neobalaenidae* or *Cetotheridae*; Fordyce and Marx 2013; Wolf et al. 2023).

### Hormones

At least nine steroid hormones (aldosterone, corticosterone, cortisol, dehydroepiandrosterone [DHEA], estradiol, estrone, estriol, progesterone, testosterone) and two thyroid hormones (thyroxine [T4], tri-iodothyronine [T3]) have been detected in baleen with immunoassays (Table 1; estriol, S. Fenstermacher, unpublished). Mass spectrometry has confirmed presence of the above steroids and several others, most notably high concentrations of androstenedione (Evey et al. 2026). The majority of published studies focus on progesterone (for its utility in identifying pregnancy) and at least one glucocorticoid (e.g., cortisol or corticosterone; for stress assessment), and multiple studies also exist for the androgens and estrogens (Table 1). Several studies utilize a comprehensive “panel” of four or more hormones on every sample (Table 1; e.g., Lysiak et al. 2018; Lowe et al. 2022; Evey et al. 2026; Shuttleworth et al. 2025; Jelincic et al. 2026a, 2026b, 2026c). No publications have yet taken full advantage of mass spectrometry’s capability for analysis of multiple steroids at once, likely due to limitations of required sample mass and cost/time considerations. Notably, steroid and thyroid hormones share commonalities of small molecular size and nonpolar behavior, and therefore other small, nonpolar molecules (e.g., certain toxicants and cytokines, among others) might be present in baleen as well, presenting opportunities for future research.

## Validations

Developing endocrine quantification techniques for any new sample type requires validations to determine the reliability and biological significance of the resulting data. Numerous reviews on other sample types describe the validation process nicely (e.g., feces; Touma and Palme 2005 , Palme 2019); hence, our goal here is not to describe all aspects of validations, but rather to give a brief methodological overview relevant for baleen. Here we focus on: (1) sampling and extraction methodology; (2) assay validations; and (3) physiological (aka biological) validations.

### Sampling and extraction methodology

The first baleen-hormone studies (Hunt et al. 2014, 2016) utilized a relatively simple extraction protocol derived from hair and feather literature, consisting of abrading a small groove on the baleen with a hand-held rotary grinder or drill, and then vortexing 100 mg of the resulting powder with 100% methanol for 2 h, followed by centrifugation, dry-down of the supernatant, and resuspension of dried hormones in 500 µl of an appropriate assay buffer. This initial technique passed validations and produced biologically plausible data, but used a large amount of sample. Shortly afterwards, Fernández Ajó et al. 2021) determined that sample mass can be reduced to 20 mg of powder, and likely to 10 mg if solvent-sample ratio is consistent across samples. Many labs now utilize 20 mg as the default sample mass (typically extracted with 4.00 mL of 100% methanol), often employing an antistatic ionizer during weighing to reduce static charge effects on apparent sample mass. Dillon et al. (2021) have further demonstrated that the amount of hormone recovered from baleen powder can be increased via incubation with a keratinase enzyme that dissolves the baleen matrix and liberates additional hormone. While keratinase may not always be necessary, it is a useful option for cases in which little powder is available, or if certain hormones are at very low concentration.

The methods described above indubitably produce useful data, yet we note that numerous questions remain regarding baleen subsampling and extraction methodology. We present some of these questions in supplementary information (Table S1), in the hope of inspiring additional research to further improve methodology.

### Assay validations

**Fig. 3:**
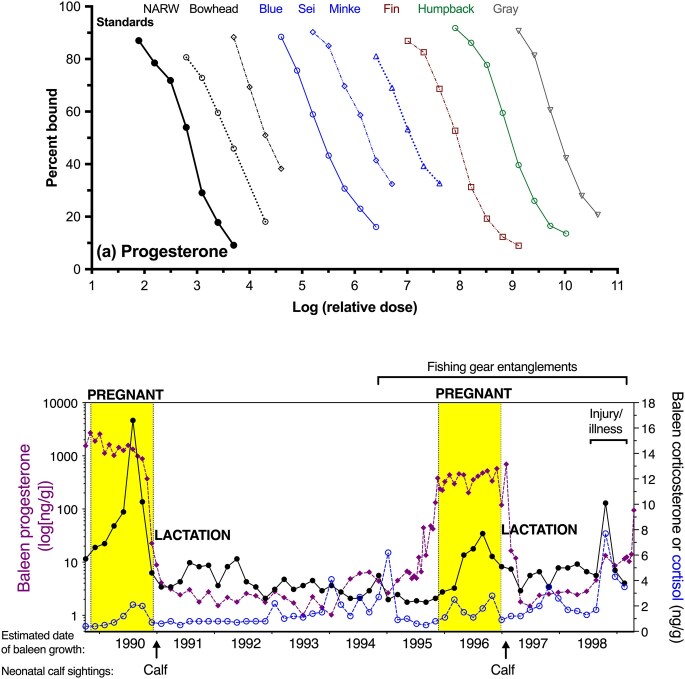
Examples of assay validations and physiological validations. Top, parallelism validation for a progesterone immunoassay tested in baleen of eight whale species; note good parallelism of progesterone standard curve (left line) to data from serially diluted baleen extracts (other lines). Bottom, physiological validation of progesterone (purple diamonds, dotted line), corticosterone (black solid circles, solid line), and cortisol (blue open circles, dashed line) assays tested on baleen of a female North Atlantic right whale. Note good correspondence of hormone elevations with known pregnancies (yellow bars) confirmed from calf sightings, and peaks of glucocorticoids corresponding with documented injury and illness. (Top panel, Hunt et al. 2017a; bottom panel, modified from Hunt et al. 2017b, used with permissionafre.)

We focus here on enzyme immunoassay (EIA, including enzyme-linked immunosorbent assay, ELISA) as this is the most common technique in baleen-hormone literature. Assay validations for EIAs typically proceed with four phases: (1) First, a brief detectability trial is performed to determine if there is sufficient binding to the assay antibody, followed by (or simultaneous with) (2) a parallelism test, which assesses the affinity with which the assay antibody binds to the presumed hormone (Grotjan and Keel 1996; King 2016; example in Fig. 3, top). Good parallelism is considered strong (though not definitive) evidence that the target hormone is indeed present; if desired, mass spectrometry can provide more conclusive evidence (e.g., Evey et al. 2026). (3) Next, an accuracy test (aka “linearity,” “matrix effect”) is performed to assess whether low vs. high concentrations can be distinguished with good mathematical accuracy at a given sample dilution, that is, despite the possible presence of interfering compounds within the sample matrix (keratin molecules, calcium salts, etc.) (Grotjan and Keel 1996; King 2016). Some of the published literature omits the accuracy validation, but we strongly recommend that it be performed for every species and analyte, as many cases exist in wildlife endocrinology literature of assays that have excellent parallelism yet demonstrate poor accuracy. (4) Finally, assay characterization tests the assay’s performance at typical sample concentrations, often including determination of the limit of detection as well as quantification of intraassay and interassay variation. Assay characterization parameters are often simply reported from the assay manufacturer’s protocols, but because assay performance can vary considerably among labs, we recommend lab-specific testing of both limit of detection and assay variation. We further recommend randomization of samples within and across assays, to minimize any effects of assay variation on longitudinal data.

Encouragingly, all hormone EIAs tested on baleen extract have passed all the above assay validations (e.g., Hunt et al. 2017; Jelincic et al. 2026a; Evey et al. 2026). Overall, baleen powder appears a “tractable” sample type that behaves remarkably well in EIAs. Nonetheless, each additional hormone and species requires new validations, and thus we encourage researchers new to endocrinology to communicate with established endocrine labs for guidance. Some additional questions of interest relevant to assay validations are listed in Table S2.

### Physiological validations

Physiological validations (aka biological validations) seek to determine whether the hormone data, however precisely they have been measured, actually correspond with the (independently known) endocrine state of the animal. In some cases, good physiological validations can even compensate for poor assay validations. For example, a progesterone assay with poor parallelism (poor assay validation) that nonetheless can correctly distinguish pregnant whales from nonpregnant whales (good physiological validation) has utility, despite the poor parallelism—though, statistical approaches and interpretation may need to be adjusted (e.g., analysis of relative patterns rather than absolute concentrations). Ultimately, the primary question is whether the data have biological meaning and can correctly identify the physiological states of interest.

Many classic physiological validations common in terrestrial research are not possible in mysticete whales, for example, removal-replacement experiments (e.g., glandular ablation or knockdown, followed by hormone supplementation), or injection of pituitary releasing hormones or radio-labelled hormones (e.g., Wasser et al. 2000; Fusani 2008, 2010). Instead, physiological validations in the large whales must proceed by means of comparing endocrine data to observed or inferred physiological state. For example, progesterone data can be compared to pregnancy status (with the hypothesis that progesterone should be highest in pregnant females), testosterone data to age class and breeding season (i.e., testosterone should be highest in adult males during the breeding season), and glucocorticoid data to known chronic stressor exposure (i.e., glucocorticoids should be elevated during entanglements in fishing gear). Thus, physiological validations for mysticete whales require already knowing something about the individual animal, typically via necropsy or sightings data (example in Fig. 3). Though such cases can be rare, even very low *n*’s can provide guidance (e.g., a single pregnant individual as in Evey et al. 2026, or a single severe entanglement as in Lysiak et al. 2018). Over time, additional cases can often be compiled so as to eventually add up to a robust physiological validation. Thus, physiological validations for large whales often play out across multiple publications over several years, with multiple research teams contributing individual case studies to a growing body of work.

For baleen hormones, physiological validations began with case studies of known pregnancies, first in bowheads with fetal presence verified at necropsy (Hunt et al. 2014), and subsequently in North Atlantic right whales, a critically endangered species with a comprehensive sightings catalog of known calving events (example in Fig. 3, bottom; Hunt et al. 2016; Lysiak et al. 2018). Baleen progesterone data in these two species demonstrated excellent correspondence to pregnancy status, with regions of baleen grown during known pregnancies having very high progesterone content. Similar patterns have since been documented in humpback (Lowe et al. 2021a), southern right (Shuttleworth et al. 2025), Rice’s (Evey et al. 2026), blue, and fin whales (A. Case, unpublished data). Several of these specimens were decades old, and recent analysis of bowhead museum specimens indicates that progesterone patterns strongly suggestive of pregnancies are detectable even in specimens over 150 years old, an exciting finding that should enable comparisons of past and present populations (Jelincic et al. 2026c).

Other hormones have also generally shown the expected patterns. For example, testosterone shows sharp and consistent seasonal peaks in adult males of bowhead, blue, and North Atlantic right whales (Hunt et al. 2018, 2022), as is expected for seasonally breeding migratory mammals—though the apparent timing of these breeding seasons has elucidated some surprises (see below). Further, adrenal glucocorticoids show elevations that correspond with known stressors, both anthropogenic (e.g., entanglement in fishing gear; Hunt et al. 2017b; Lysiak et al. 2018; Rolland et al. 2019; Lowe et al. 2021; Evey et al. 2026; plastic ingestion with internal injury, Evey et al. 2026) and natural (e.g., reproduction-related stress [Jelincic et al. 2026a, 2026b, 2026c; Hunt et al. 2017b]; chronic or infectious disease [Hunt et al. 2017b; Gabriele et al. 2021 ]; cutaneous wounds [Fernández Ajó et al. 2020]). Thus, physiological validations to date have uniformly been excellent for the steroid hormones.

The thyroid hormones, however, have not yet been fully physiologically validated as of this writing. Thyroid hormones regulate developmental processes, thermogenesis, and resting metabolic rate in mammals (Behringer et al. 2018; Zwahlen et al. 2024). The common hypothesis for patterns of thyroid hormones in adult whales is typically that T4 and T3 will be lower during periods of nutritional stress, representing a predicted lowering of metabolic rate to conserve energy during fasting and starvation (Wasser et al. 2010; Martinez and Ortiz 2017; Behringer et al. 2018). This theoretical framework implies that the thyroid hormones should generally exhibit negative correlations with adrenal glucocorticoids. However, contrary to these predictions, available baleen data indicate that T3 is either positively correlated with adrenal hormones (bowhead whale, Hudson et al. 2025, Jelincic et al. 2026a; southern right whale, Fernández Ajó et al. 2020) or exhibits no correlation (Jelincic et al. 2026a). Lysiak et al. (2018) documented one case of elevated thyroid hormones co-occurring with emaciated body condition in an entangled North Atlantic right whale, but it has not yet been possible to discriminate the possible metabolic effects of entanglement (e.g., energetic burden of dragging gear) from those of emaciation *per se*. Thus, few data exist on patterns of thyroid hormones in whale baleen in relation to independently confirmed nutritional status, likely due to lack of relevant datasets on nutritional status of individually known whales from which baleen specimens also exist. It thus remains an open question what thyroid hormones may actually tell us about mysticete whale physiology. In future studies, pairing of baleen thyroid-hormone data with measurements of blubber thickness from necropsies, or with any ante-mortem data that may exist on body condition during life (e.g., from photogrammetry), would be most informative. Given the recent occurrence of unusual mortality events and poor reproduction thought to be related to nutritional stress (e.g., gray whales, Christiansen et al. 2021; North Atlantic right whales, Christiansen et al. 2020), as well as dramatic shifts in prey species with ongoing marine climate change (Sadykova et al. 2020; Bas et al. 2025), we encourage further investigation into thyroid hormones in baleen, as well as other biomarkers that might be indicative of nutritional stress.

## Individual case studies

Since its earliest inception, much of the baleen-hormone literature has entailed a series of descriptive studies, with each publication typically combining some of the above validations with a small number of individual case studies (Table 1). Each individual case study typically comprises dozens of samples (sometimes over 100) spanning years (i.e., repeated-sample design), often with multiple hormones as well as bulk isotope analyses, across numerous physiological events. In sum, each case is a complex story of individual physiology over time, and thus, these individual-whale datasets tend to be published at a slow rate, often with an *n* of just 1–4 whales per paper (Table 1). This is largely a consequence of the high number of samples per whale, and associated labor and funding constraints. Such papers often have a stated goal of providing essential descriptive information, such as hormone ranges, baselines and peaks compared across sexes, ages, and reproductive states. Descriptive data of this sort will be necessary for each hormone, species and age class, and provide critical information that lays the groundwork for future research.

However, descriptive studies do not merely provide baselines and ranges. Notably, almost every descriptive study has also discovered unexpected patterns that have revealed important new findings about cetacean physiology. One prominent example is the unexpectedly lengthy gestation reported in all *Balaenidae* yet studied (bowhead, North Atlantic right, and southern right). All three of these species exhibit elevations of progesterone in baleen lasting up to 22 months (Hunt et al. 2017; Lysiak et al. 2023; Shuttleworth et al. 2025; Jelincic et al. 2026c), substantially longer than previous estimates of ∼11–13 months of gestation (Best 1994; Reese et al. 2001; Tarpley et al. 2021). While some uncertainty remains (i.e., temporal resolution of baleen data; see Shuttleworth et al. 2025), this finding has had important implications for understanding female fecundity and associated limits on population growth rate. For example, the long-hypothesized “resting year” demonstrated by all female *Balaenidae* (i.e., a three-year calving cycle assumed to represent one year of pregnancy, one year of lactation, and one year of “rest”; reviewed in Kraus et al. 2007), may not be a resting year at all, but rather the first year of a very long pregnancy. Similarly, testosterone peaks of males often appear to occur at different months of the year than expected (e.g., Hunt et al. 2022), corroborating the extended gestation of the females (i.e., initiation of pregnancy in females aligns with timing of testosterone peaks in males). As these are migratory species, this further implies that not just the timing of breeding but also the location of breeding grounds may have been misidentified in the past, a finding with obvious conservation implications. Additional surprises include dramatic variation in female intercalving interval (i.e., Jelincic et al. 2026c, Allison Case personal communication), unexpectedly high rates of pregnancy loss (e.g., Lysiak et al. 2023; Jelincic et al. 2026c), possible reproductive senescence (Hunt et al. 2022; Jelincic et al. 2026a), occurrence of testosterone cycling in very small (young) males (Hunt et al. 2022), a “moribund pattern” of elevations in all hormones—including reproductive hormones—in chronically stressed whales (Evey et al. 2026), unexpectedly high hormone concentrations in calves (Evey et al. 2026), differing patterns in the two glucocorticoids (Hunt et al. 2017b; Jelincic et al. 2026a, 2026b, 2026c), and general higher baleen concentrations of corticosterone than cortisol (Hunt et al. 2017b; Brown et al. 2026; Evey et al. 2026, Jelincic et al. 2026a, 2026b, 2026c). Given the importance of such findings for basic cetacean biology as well as implications for conservation and management, baleen-hormone case studies provide a powerful example of the value of descriptive science. We therefore encourage continued attention to detailed presentation of individual case studies, as well as to the essential descriptive analyses needed for each mysticete species.

## Bigger questions, more whales: the path forward

As one validation after another is completed, baleen-hormone research as a field has increasingly been able to look beyond validations and case studies to address hypothesis-driven questions of broader ecological significance, that is, shifting from “what are the patterns” to “what is driving the patterns.” Broadly, such research efforts seek to understand how oceanographic, anthropogenic, and intrinsic factors affect cetacean physiology and life history transitions (nonpregnant to pregnant, immature to mature, etc.). For such efforts, more individual whales will be needed to disentangle individual variation and random effects from associations with major variables of interest. An early example of this approach is (Fernández-Ajó et al. 2020) examination of patterns in glucocorticoids in 36 southern right whale calves (Argentina population), in which adrenal stress hormones were strongly correlated to the degree of wounding experienced by the calves before death, suggesting a specific cause of death (attacks by kelp gulls, *Larus dominicanus*) during an unusual mortality event in this population. This study took advantage of the short length of calf baleen to study many more individuals than is usually possible, and in fact set a record for sample size of whales that is unmatched to this day. Studies are now in progress with comparable or greater sample sizes (blue and fin whales, A. Case, unpublished; bowhead whales, J. Jelincic, unpublished) to examine effects of oceanographic regime shifts, anthropogenic stressors (e.g., commercial whaling), loss of arctic pack ice and other impacts of climate change, and impacts of emerging contaminants. This phase of research is likely to produce findings relevant not just to whales but more broadly to long-lived mammals both marine and terrestrial.

As such datasets grow larger, they will require adjustments in study design, including robust data management and statistical frameworks that can handle such data. Numerous statistical techniques exist for time-series data generally, and Reed et al. (2026, this volume) presents several approaches to time series analyses that are appropriate for baleen endocrine data. Additionally, the structure of these data means that some techniques previously used for human studies (e.g., hierarchical state-space models; Liu et al. 2014) can be utilized to study the complex relationships between hormones, as well as populations and individuals, in greater detail. Larger *n* also facilitates the extension of baleen research findings beyond individual case studies to examine likely effects on populations. For example, the PCoMS (Population Consequences of Multiple Stressors) modeling framework, originally applied primarily to marine mammal behavioral and photographic data, increasingly incorporates physiological data, and provides a powerful tool via which the multi-year individual datasets characteristic of baleen hormone studies can be extended to the population level (New et al. 2026, this volume).

However, one logistical hurdle of this exciting new phase of research is the work required by each baleen specimen. To take the long baleen of bowheads as an example, just eight adults—a modest sample size for most physiological studies—can easily sum to over 1200 subsamples of baleen powder, each specimen producing complex individual profiles of multiple hormones with numerous variables and a pronounced temporal component. Literature indicates that analysis of even a single adult baleen specimen can entail years of labor (Table 1). It could therefore take decades to compile datasets with the sample sizes necessary to address the types of questions outlined above. Given the rapid pace of change in marine environments and the pressing conservation issues facing cetacean populations today, it would be ideal to accelerate progress. The time may therefore be ripe to consider a baleen-hormone research consortium or data repository, such that data can be compiled and shared across research teams. Such a consortium could also minimize impacts of destructive sampling on rare specimens, that is, by eliminating redundant sampling as well as via communication of improved methodologies. Similar research consortiums and repositories already exist for other types of data, some involving cetaceans (e.g., Pacific Coast Gray Whale Consortium, pcfgconsortium.org; North Atlantic Right Whale Consortium, narwc.org) as well as other fields of science such as cell-line consortiums and genetic data repositories. Such an endeavor will require considerable thought as to how best to organize the data, track methodology, and address questions of interlab variation (a paramount concern for hormone data; Fanson et al. 2017), and, not least, ensure appropriate credit for contributions and thus encourage participation. A baleen-hormone research consortium will take effort and widespread willingness across the field to adopt a collaborative mindset. The payoffs, however, may be considerable in the form of greater sample sizes than any team could produce alone, a more rapid pace of research, and, hopefully, more impact on real-world conservation and management of the world’s great whales.

## Conclusions

Whale baleen constitutes a unique endocrine sample type that can reveal biologically meaningful patterns of reproductive, stress, and metabolic hormones across multiple years, in some cases multiple decades, yet with sufficient temporal resolution to discriminate seasonal effects, examine annual cycles, and investigate temporal relationships of stressors and reproductive events. The stability of baleen steroid and thyroid hormones promises capability to compare past populations with recent and modern populations of whales, providing a rare opportunity to examine influences of numerous anthropogenic and environmental factors across different historic eras. While many methodological questions remain to be answered, validations have largely been successful, albeit with certain gaps still deserving of attention (e.g., pygmy right whale, thyroid hormones). Detailed individual case studies have provided essential descriptive information on ranges, baselines and common endocrine patterns in various species and demographic groups, while also revealing numerous unexpected findings relevant for basic whale biology as well as for conservation applications. We encourage continued attention to descriptive case studies and methods optimization, while also expanding goals, aims and study designs so as to support hypothesis-driven research of broad ecological relevance. Doing so will require increasing the number of individual whales studied, a task that may prove more tractable with the adoption of organized collaborative approaches. As illustrated by the fecal hormone field four decades ago, it takes more than a decade, and many research teams, to validate and launch a new technique. With this in mind, baleen hormone research has made considerable progress in its first dozen years. With continued cooperation and investigation, baleen hormone research as a field is likely to continue to expand its reach, providing unique and important insights into the biology of the world’s largest and perhaps most fascinating predators, the mysticete whales.

## Author contributions

K.E.H, J.J., A.C., and R.E. conceived the idea; K.E.H., L.N., and C.L.B. secured funding; K.E.H. and J.J. wrote the first draft of the manuscript; J.R., L.N., N.S., and D.D. contributed edits and original text to the initial draft; all authors contributed substantively to writing and editing of the final manuscript.

## Supplementary Material

icag063_Supplemental_File

## Data Availability

All relevant data are presented in the text, figures, and tables.

## Notes

From the symposium “Hormones at sea: New frontiers in marine mammal endocrinology and ecophysiology” presented at the annual meeting of the Society for Integrative and Comparative Biology, January 3-7th, 2026.
