# Supplementary material for: A Dozen Years of Baleen Hormones: Validations, Discoveries, Surprises, and Next Steps
Source: Integr Comp Biol. 2026 Jun 8;66:icag063. doi: 10.1093/icb/icag063 (PMC13403903; doi:10.1093/icb/icag063)
Supplement: icag063_Supplemental_File [file icag063_supplemental_file.docx]

**SUPPLEMENTARY INFORMATION**

Table S1. Some unanswered questions regarding baleen hormone methodology, data, and interpretations.

| **BALEEN GROWTH RATE (BGR)** |
| --- |
| - Isotope and sightings data suggest BGR is consistent on a seasonal scale, but is it also consistent at finer time scales? (e.g. week to week) - Is BGR affected by physiological status (e.g., stress, body condition, reproductive state)? - Given that some hormones influence epidermal activity (e.g. thyroid hormones), can hormones themselves directly affect BGR? - How might we evaluate these questions? Can further synthesis of sightings data, isotope data and hormone data provide guidance? |
| **BALEEN SAMPLING** |
| **Intra- and inter-plate variation**   - Do different baleen plates from the same whale produce the same patterns of hormones? (e.g., adjacent baleen plates, left vs. right side of mouth, anterior vs. posterior surface of same plate, short vs. long baleen from same whale) - Do baleen plates from the left vs. right sides of the mouth exhibit any additional differences in those species that exhibit asymmetrical feeding behaviors (e.g., fin whales)? |
| **Sampling depth**   - Does sampling depth (e.g.*,* drilling into medulla) affect the hormone profile of a given baleen plate? - Does the erosion of the plate tip preferentially remove baleen cortex, such that the plate's timeline may shift toward the tip of the plate? |
| **Pulverization and particle size:**   - Does the particle size produced by a hand-held grinder affect apparent hormone content, and does this particle size change with sampling technique (e.g.*,* pressure of drill bit) or along the length of the plate? - Do other grinding techniques, or a two-step pulverization process (e.g., dremel, then bead blaster) produce improved data? |
| **Base-of-plate and tip-of-plate effects:**   - Why do the proximal-most point and distal-most point often both have unusually high hormone concentrations? Is this phenomenon a particle-size effect, or is it fully attributable to the "small sample effect" (spuriously high apparent hormone concentration in samples of very small mass; Hayward et al. 2010; Fernández Ajó et al. 2022)? Should these two points be routinely excluded from analysis? - Why do all hormones typically increase at the base of the plate in moribund whales near death ("moribund effect")? |
| **Temporal resolution:**   - What is the temporal resolution of a given point on a baleen plate? - How does temporal resolution change with species, age or baleen growth rate? - Do gradual "ramp-up" and "ramp-down" of hormone peaks (e.g.*,* starts and ends of pregnancies) reflect biological reality, or are such patterns a result of "running averages" due to broad temporal resolution? - What approaches could answer these questions? |
| **EXTRACTION** |
| **Minimum sample mass**   - Can sample masses be reduced below 10 mg? What changes in weighing protocols might be necessary for tiny sample masses? - What causes the "small sample effect" reported for all powder-type samples (e.g., fecal powder, feather powder), wherein small samples have spuriously inflated hormone content (Hayward et al. 2010; Fernández Ajó et al. 2022)? Can the small sample effect be eliminated? |
| **Extraction solvent**   - Would non-alcohol solvents (e.g., ethyl acetate, ethyl ether, dichloromethane) improve hormone yield or hormone patterns? - Would more complex extraction methods (e.g.*,* double extraction) improve hormone yield or clarify hormone patterns? - Conversely, would additional extraction steps have negative effects, e.g. pipetting losses or increased variation in resulting data? |
| **Extraction time**   - Minimum time: Can the standard 2-hour vortex time be reduced? - Maximum time: Is there a "maximum time" beyond which longer times produce no further change? |
| **Extraction temperature**   - Does temperature during extraction or during dry-down affect hormone concentrations? Specifically, can the common bottleneck of dry-down be accelerated by means of higher temperatures without affecting data? |
| **Percentage of hormone recovered ("recoveries")**   - Is there a method by which the percentage of native hormone recovered from baleen powder can be assessed? (i.e., given that pure hormone experimentally added onto a baleen surface presumably will not behave like native hormone embedded within keratin) - Is it valid to assume that percentage-recovery, even if not known, is similar across samples? |
| **SPECIES** |
| - Will baleen hormone techniques work for the pygmy right whale, given its evolutionary distance from other rorquals? - Will baleen hormone techniques work for close relatives of species already tested? (e.g., North Pacific right, Bryde's whale group) - Can baleen hormone techniques be extended to linear, continuously growing, hard-part keratin structures of other mammals? |
| **ANALYTES & BIOMARKERS** |
| **Steroid hormones:**   - What other steroid hormones might be present and worthy of study? - Is it useful to study all hormones possible, or is there a smaller panel of "most informative hormones"? - Do baleen hormones represent the "free" (unbound to carrier proteins) form of steroid hormones that circulate in plasma, as opposed to "total" (free + bound) circulating hormone? |
| **Thyroid hormones:**   - Are T3 or T4 a reliable marker for nutritional status? How might this be tested? - Would a larger panel of thyroid hormones (e.g., assaying both T4 and T3, and/or potentially adding reverse-T3 or other forms) be more informative than a single thyroid hormone? |
| **Other hormones and signaling molecules:**   - Are any peptide or protein hormones, cytokines, or other monoamines (e.g., beyond the thyroid hormones) detectable in baleen? |
| **Stable isotopes:**   - Must isotopes be analyzed for every baleen plate? How much inaccuracy is introduced by estimating a baleen plate's timeline by means of species- or age-specific averages for baleen growth rate? - Can hormone-assay pellets (after centrifugation) be subsequently utilized for isotope analyses? - To what degree can environmental change affect isotope cycles, e.g. via changes in prey distribution, selection, or migration behavior? Do such changes affect the assumptions underpinning temporal interpretation of isotope data (e.g. one isotope cycle = one year)? - How might compound-specific isotope analyses best be added to baleen-hormone research, given typical budget limitations? |
| **Additional biomarkers and analytes:**   - Can toxicology be routinely performed on the same samples utilized for isotope and endocrine analyses? - Can the required sample mass (often 200 mg) for common toxicology analyses be reduced? - What other physiological biomarkers are detectable in baleen? Can a biomarker indicative of successful parturition, lactation, or weaning be identified? Can biomarkers useful for assessing immune function or estimating age of whale be identified? - Does macroanatomy, microanatomy, or molecular composition of baleen (e.g. visually apparent "growth ridges", external stress resistance, keratin protein composition) provide additional information relevant for assessment of health and physiology (parallel to veterinary information derived from mammalian hair, hoof, etc.)? - Can baleen specimens collected in unknown years be dated (e.g.*,* to nearest decade)? - How might genetic analyses best be utilized to complement baleen endocrine research? What methods could overcome difficulties commonly reported in extracting sufficient DNA from baleen for certain desired analyses (species id, sex id, etc.) ? |
| **DATA ANALYSIS** |
| - How might the trophic ecology information also produced by isotope analyses be best integrated with endocrine information? - What changes or new approaches in data management and statistical analyses may be necessary to handle large-*n* baleen datasets? - Could techniques for longitudinal data analyses previously developed for human studies or terrestrial wildlife be ported to cetacean baleen data? - Can baleen physiological data be utilized in population modeling approaches that have not previously incorporated this type of data, i.e. to extend findings to the population level? |
